# Expression of urinary exosomal miRNA-615-3p and miRNA-3147 in diabetic kidney disease and their association with inflammation and fibrosis

**DOI:** 10.1080/0886022X.2022.2121929

**Published:** 2023-01-25

**Authors:** Jiaxin Wang, Yiying Tao, Fan Zhao, Tong Liu, Xiahong Shen, Ling Zhou

**Affiliations:** Department of Nephrology, The First Affiliated Hospital of Soochow University, Suzhou, China

**Keywords:** Exosomes, microRNA, diabetic kidney disease, inflammation and fibrosis

## Abstract

**Background:**

Diabetic kidney disease (DKD) is one of the most common chronic complications of type 2 diabetes mellitus (T2DM), and it is particularly important to identify a high-quality method for evaluating disease progression. Urinary exosomes contain microRNA that might promise early diagnostic and monitoring markers of DKD. The present study aimed to identify novel exosome-related markers associated with inflammation and fibrosis to assess the progression of DKD.

**Method:**

Exosomes were extracted from the urine of 83 participants to determine the expression levels of miRNA-615-3p and miRNA-3147 in 20 healthy people, 21 patients with T2DM and 42 patients with DKD, as determined by RT-qPCR. The circulating expression level of TGF-β1 was detected by ELISA. Serum Cystatin C was measured by a latex-enhanced immunoturbidimetric method. The correlation analyses were performed for all clinical and laboratory parameters.

**Result:**

The expression level of urinary exosomal miRNA-615-3p in DKD patients was significantly higher than that in the control group and the T2DM group by RT-qPCR. The expression of miRNA-3147 showed an upward trend in the three groups of subjects, but it was not statistically significant. The urinary exosomal miRNA-615-3p was positively correlated with serum Cystatin C, plasma TGF-β1, creatinine, BUN, PCR and 24-h urine protein, and negatively correlated with eGFR and albumin. The diagnostic efficacy of urinary exosomal miRNA-615-3p combined with the ACR was higher than that of ACR alone.

**Conclusions:**

Urinary exosomal miRNA-615-3p may be used as a novel biomarker for evaluating the progression of DKD, and may be involved in the process of inflammation and fibrosis in DKD. The combined diagnosis of urinary exosomal miRNA-615-3p and ACR may be used as more stable and sensitive diagnostic criteria for DKD.

## Introduction

Diabetes is a major public health challenge worldwide [1], of which type 2 diabetes mellitus (T2DM) accounts for about 90% [2], and it is the most common cause of chronic kidney disease (CKD) and end-stage renal disease (ESRD). It is often insidious and difficult to detect, leading to an increase in the incidence of various acute and chronic complications, particularly cardiovascular disease. T2DM patients are more likely to develop chronic kidney disease than non-diabetic patients [3], developing in approximately 20–40% of patients with T2DM. The pathogenesis of DKD generally includes four aspects, hemodynamic abnormalities, inflammatory response, fibrosis, and metabolic abnormalities [4,5]. Numerous studies have documented that inflammation and fibrosis are among the most important links in the pathogenesis of DKD [6]. In a high-glucose environment, excess oxygen free radicals accumulate in kidney tissue, resulting in oxidative stress [7]. It can damage endothelial cells, stimulate the release of related inflammatory factors into the blood, such as Cystatin C (Cys-C), interleukin-6 (IL-6), tumor necrosis factor-α (TNF-α), transforming growth factor-β1 (TGF-β1) and vascular cell adhesion molecules-1 (VCAM-1) [8]. These inflammatory mediators promote the development of inflammatory responses, activate molecular pathways such as TGF-β1-Smad2/Smad3, and induce extracellular matrix (ECM) production and deposition in the tubulointerstitium [9], resulting in extensive renal tissue fibrosis.

Exosomes are membrane-bound extracellular vesicles secreted by cells, that contain various constituents such as protein, DNA and RNA. Interest and research focused on exosomes have dramatically increased, recognizing the intercellular crosstalk of cell-to-cell communication that could result in changes in cellular and biological functions of the recipient cells. The nucleic acids that they carry have been implicated in the pathophysiological process of kidney diseases [10], including acute kidney injury (AKI), vasculitis [11], and chronic kidney disease (CKD). The one of principal nucleic acids involved in regulating molecular pathways are miRNAs [12]. They are non-coding RNAs containing about 18–22 nucleotides, that serve as important post-transcriptional regulators of fundamental biological pathways such as cell differentiation, apoptosis and cell proliferation [13]. Wang et al. [14] reported that, under a high glucose environment, miRNA-21 in renal tubular epithelial cells could accelerate the deposition of ECM and induce the expression of α-SMA by activating the TGF-β1/Smads signaling pathway. Similarly, other microRNAs have been implicated in the pathogenesis of DKD involving renal tissue fibrosis and inflammation, such as miRNA-214 [15], miR-23a-3p [16], etc. In conclusion, miRNAs have a correlation with the inflammatory response and fibrosis of DKD.

Compared with free miRNAs in urine, miRNAs carried by urinary exosomes are more stable and are not affected by Ribonuclease (RNase) [17,18]. In recent years, with the in-depth study of urinary exosomal miRNAs and the popularization of gene detection technology, more and more miRNAs have been confirmed to be abnormally expressed in samples from DKD patients, such as miRNA-4534 [19], miRNA-188-5p [20], miRNA-150-3p and CCL21 mRNA [21], etc. However, most of these differentially expressed miRNAs have not been verified by RT-qPCR. Our previous study showed that miRNA-3147 and miRNA-615-3p were differentially expressed in the circulating exosomes of DKD patients. (In a previous, as yet unpublished, study using small RNA screening and RT-qPCR, our group showed that plasma exosome miRNA-3147 and miRNA-615-3p were differentially expressed in DKD patients) Therefore, based on extracting reliable urinary exosomes [22], the present study aimed to determine the clinical value of urinary exosome-derived miRNA-615-3p and miRNA-3147 in diagnosing DKD and assessing disease progression, with an ultimate goal of preventing the occurrence of DKD and providing a theoretical basis for early intervention.

## Materials and methods

### Patient samples

This study comprised 21 patients with T2DM and 42 patients with DKD who were treated in the First Affiliated Hospital of Soochow University from June 2019 to December 2021 for the preparation of urine and blood samples. Additionally, during the same period, 20 healthy subjects (derived from our hospital medical staff) were recruited as the control group (N). Guidelines published in 2010 by the American Diabetes Association were employed as the diagnostic criteria for T2DM patients, and The National Kidney Foundation Kidney Disease Outcomes Quality Initiative (NKF-KDOQI) guidelines were used as the diagnostic criteria for selecting patients with DKD. Other causes of CKD including glomerular, tubular, and renal interstitial diseases, autoimmune diseases, blood diseases, malignant diseases, acute complications of diabetes and morbid obesity (BMI ≥ 40 kg/m2) were excluded. The clinical data for these 83 patients were collected, including age, gender, body mass index (BMI), mean arterial pressure, glycosylated hemoglobin (HbA1c), blood glucose, low-density lipoprotein-cholesterol (LDL-C), high-density lipoprotein-cholesterol (HDL-C), blood urea nitrogen (BUN), creatinine, total cholesterol (TC), triglyceride (TG), 24-h urine protein, urine protein-to-creatinine ratio (PCR), urine albumin-to-creatinine ratio (ACR), alkaline phosphatase (ALP), albumin, globulin, Glutamyl transpeptidase (γ-GT), neutrophil/lymphocyte ratio (NLR), platelet/lymphocyte ratio (PLR), lymphocyte/monolymphocyte ratio Nucleocytosis ratio (LMR) and monocyte/high-density lipoprotein ratio (MHR). All specimens were obtained with the consent of the patients themselves and were approved by the Clinical Research Ethics Committee of our hospital. Ethical review number: (2020) Lun Research Approval No. 196.

### Isolation and characterization of urinary exosomes

From all study participants, 5 mL of anticoagulated venous blood and 15 mL of fasting morning urine were collected. Exosomes were extracted from urine samples by high-speed centrifugation, ultrafiltration enrichment and ExoQuick™ Exosome Precipitation Kit (EXOTC50A-1, SBI System). Briefly, urine was subjected to continuous centrifugation at 3500 × *g* for 15 min and 12,000 × *g* for 30 min to remove blood cells, dead cells and cell debris to obtain supernatant, which was then enriched using an ultrafiltration centrifuge tube (15 mL, 100KD, MWCO). We obtained approximately 3 mL of enriched urine sample, and finally added ExoQuick™ exosome precipitation reagent in a ratio of 5:1. After standing upright at 4 °C for 12 h, it was centrifuged continuously at 1500 × *g* for 30 min and 1500 × *g* for 5 min, to obtain the exosomes. The pellet was resuspended in 1 × PBS for later use. Urinary exosome characterization was performed using a transmission electron microscope (TEM) for morphology, nanoparticle tracking analyzer (NTA) for particle size analysis, and Western blotting (WB) for signature protein identification. For morphological observation, exosomes resuspended in 1 × PBS were fixed with 2% paraformaldehyde (PFA) at a ratio of 1:1, placed on a transmission electron microscope grid until dry, followed by 1% glutaraldehyde fixation, purified water washing and 1% phosphotungstic acid (PTA) staining. The prepared samples were observed using TEM (FEI Corporation, Hillsboro, OR, USA). For particle size analysis, we diluted the exosomes with a 0.22 μm filtered PBS solution, injected 0.5 mL of double distilled water into the sampling plate of the laser module for debugging, removed the laser module and cleaned it again. The diluted exosomes were injected into the sampling plate with a 1 mL syringe and analyzed using NanoSight LM10 (Malvern Panalytical, UK) to calculate the size concentration of exosomes. The marker proteins, exosomes, and exosome-depleted supernatants (EDS; negative control) from T2DM patients, DKD patients, and healthy subjects were extracted for analysis. Added together were 30 μg of exosomes and the same volume of EDS for SDS-PAGE electrophoresis, PVDF membrane transfer, nonfat milk blocking, primary antibody incubation, secondary antibody incubation, and chemiluminescence exposure. ALIX and GM130 primary antibodies were purchased from Cell Signaling Technology (Cat. No. 74220), and anti-CD63 was purchased from Abcam (Cat. No. ab252919).

### Extraction of EV-derived RNA

Following the manufacturer’s instructions, total RNA was extracted from exosomes using an RNA-easy reagent. After total RNA was quantified by NanoDrop2000 (Termo Fisher, CA, USA), HiScript III All-in-one RT SuperMix Perfect for qPCR (Vazyme, Nanjing, China) and SYBR Green qPCR Mix (Beyotime, Nantong, China) were used for reverse transcription and real-time fluorescence quantification, respectively (RT-qPCR). The relative expression levels of miRNA-615-3p and miRNA-3147 in urinary exosomes were detected by polymerase chain reaction. Based on previous publications, U6 was chosen as the internal control. miRNA-615-3p: Forward: 5′-CGTCCGAGCCTGGGTCTC-3′, Reverse: 5′-AGTGCAGGGTCCGAGGTATT-3′; miRNA-3147: Forward: 5′-GGGTTGGGCAGTGAGGAGG-3′, Reverse: 5′-AGTGCAGGGTCCGAGGTATT-3′; U6: Forward: 5′- GCTTCGGCAGCACATATACTAAAAT −3′, Reverse: 5′-CGCTTC ACGAATTTGCGTGTCAT −3′.

### Determination of plasma TGF-β1 and serum cystatin C

From all participants, 5 mL of EDTA anticoagulated venous blood was collected and the venous blood was centrifuged at 3500 × *g* for 15 min to remove blood cells, dead cells and cell debris, and the supernatant liquid was aspirated. The supernatant obtained from the separation is the plasma, and it was subjected to enzyme-linked immunosorbent assay (ELISA) to determine the expression levels of TGF-β1 in plasma by using Human TGF-β1 ELISA Kit (Linktech, Hangzhou, China). In addition, serum samples from subjects were collected to determine the expression of serum Cystatin C. Cystatin C was analyzed on the Siemens ADVIA 2400 automatic biochemical analyzer, using the latex-enhanced immunoturbidimetric method.

### Statistical analysis

Statistics and graphs were performed using SPSS 20 statistical software (SPSS, IBM, Armonk, NY, USA) and GraphPad Prism 8.0.1 (GraphPad, San Diego, CA, USA). Continuous variables were described as mean ± standard deviation (SD) or medians, and categorical variables were described by counts and percentages. One-way ANOVA、Kruskal-Wallis rank-sum test was used for three groups of continuous variables, and *t* test, Chi-Square test and Mann-Whitney *U* test were used for two groups of continuous variables. Pearson correlation analysis and Spearman correlation analysis were used to analyze the correlation. The diagnostic value was calculated using the receiver operating characteristic curve, ROC) and area under the curve (Area under curve, AUC). *p* < 0.05 was considered statistically significant.

## Results

### Characterization of extracellular vesicle (EV) in T2DM, DKD patients and control subjects

**Figure 1.**
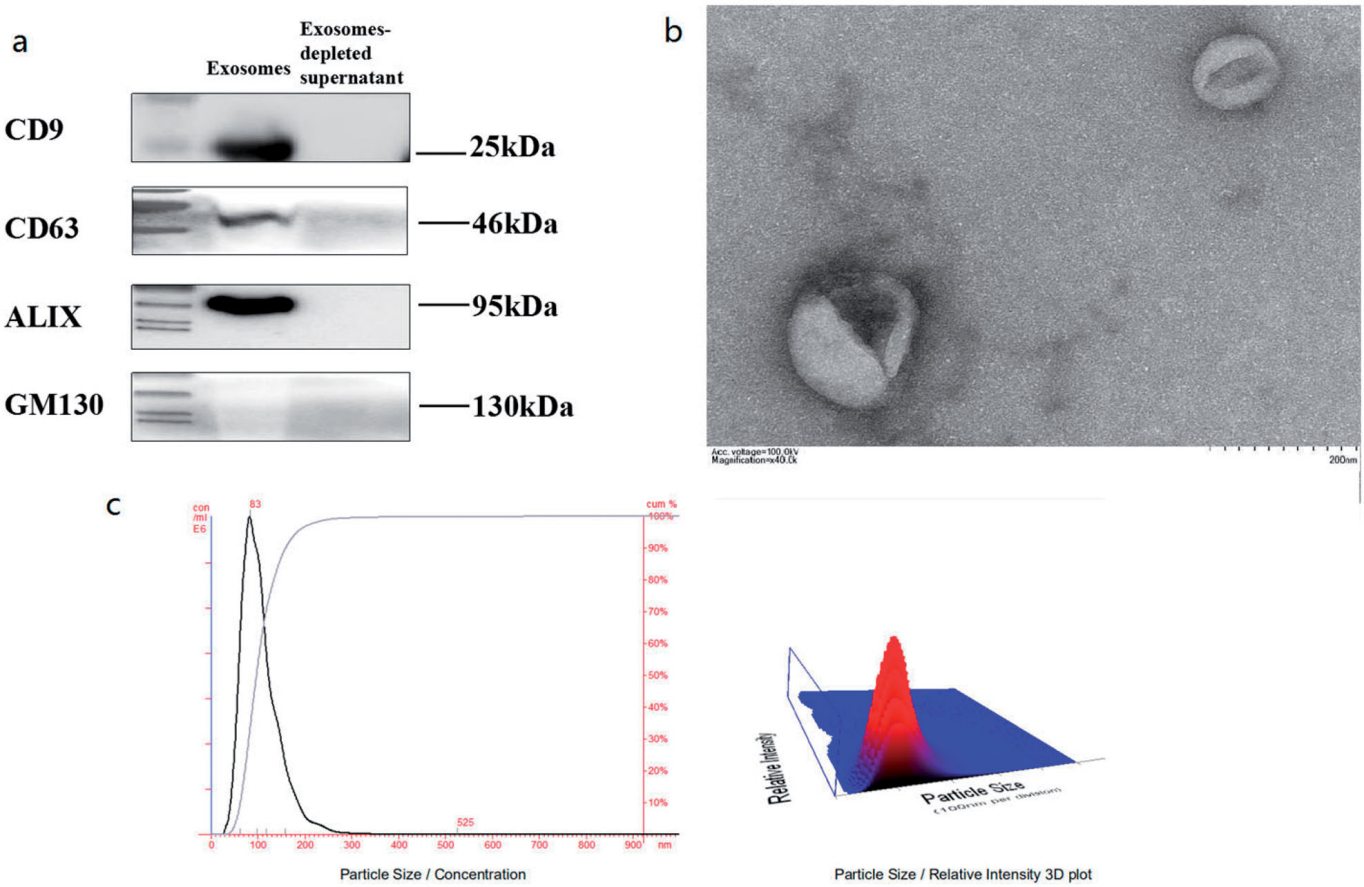
Characteristics of extracellular vesicle (EV) in subjects. 15 mL of morning urine from subjects was collected. Extracellular vesicles were extracted from urine by high-speed centrifugation (12000 × *g*, 30 min), ultrafiltration enrichment (100KD, 15 mL) and ExoQuick™ Exosome Precipitation Kit (EXOTC50A-1, SBI System) Resuspend in 1x PBS and store in −80 °C freezers until use. (a) WB results for exosome marker evaluation; PBS served as a negative control. (b) Electron Microscopy of Exosomes. (c) Particle size analysis of exosomes by nanoparticle tracking analyzer.

According to electron microscopy, nanoparticle tracking analyzer and WB results (shown in Figure 1), extracellular vesicle (EV) in control subjects, T2DM patients and DKD patients had diameters ranging from 40 to 160 nm; most were approximately 100 nm. It was consistent with previous reports. The shapes were round and oval, with a double-layer membrane structure, with the middle dyeing darker, showing a typical “saucer-like” appearance. The size distribution of exosomes ranged from 63 to 159 nm, with a peak size of 80 nm. Anti-CD63 and ALIX antibodies were positive in isolated exosomes, while GM130 was not significantly expressed in the exosomes.

### Analysis of the expression levels of miRNA-615-3p and miRNA-3147 in urinary exosomes

**Figure 2.**
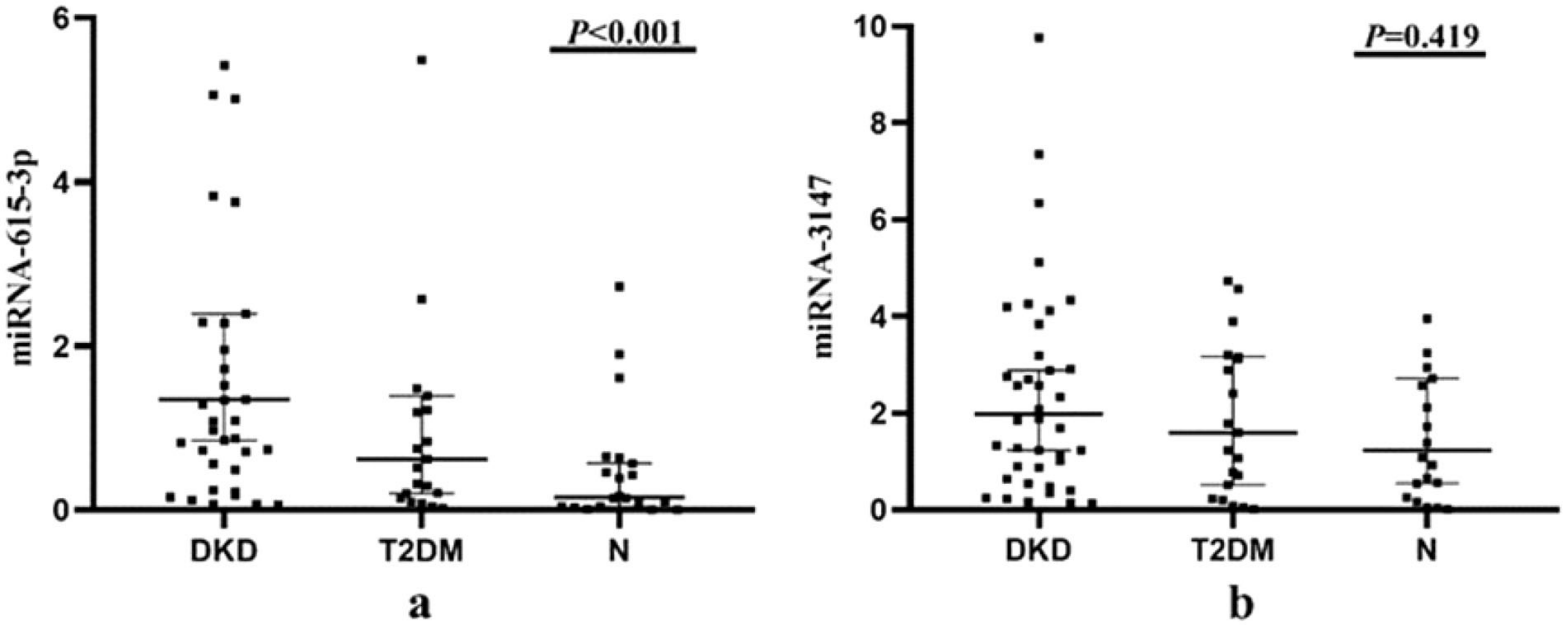
Expression levels of miRNA-615-3p and miRNA-3147 in urinary exosomes. DKD, T2DM and N represent the DKD group, T2DM group and control group, respectively. (a) Expression levels of urinary exosomal miRNA-615-3p in three groups of patients. (b) Expression levels of urinary exosomal miRNA-3147 in three groups of patients.

We collected clinical data on 83 subjects (shown in Table 1). The expression levels of miRNA-615-3p and miRNA-3147 derived from urinary exosomes in all three groups were determined by RT-qPCR (shown in Table 2 and Figure 2). We analyzed the obtained data using SPSS 20 statistical software (SPSS, IBM, Armonk, NY, USA). Compared with the N group, miRNA-615-3p in the urinary exosomes of the T2DM group and the DKD group were up-regulated. In parallel, the expression of miRNA-615-3p in urinary exosomes in the DKD group was significantly up-regulated compared with the T2DM group (*p* < 0.001). The expression level of urinary exosome-derived miRNA-3147 in the T2DM group was up-regulated compared with the N group, but the difference was not statistically significant (*p* = 0.419).

**Table 1.** Clinical characteristics of the included subjects.

Clinical parameters	*N*	T2DM	DKD	*P*-value
	(*n* = 20)	(*n* = 21)	(*n* = 42)	
Gender (male, %)	14 (70.0%)	10 (47.6%)	28 (66.7%)	0.249
Age (years)	44.65 ± 13.60	55.33 ± 13.33	61.57 ± 11.90	<0.001
BMI	24.03 ± 2.66	24.37 ± 3.47	25.75 ± 0.66	0.177
Mean arterial pressure (mmHg)	100.00 ± 1.83	100.81 ± 2.48	105.93 ± 1.84	0.078
HbA1c (%)	4.90 (4.25–5.58)	8.50 (7.30–10.80)	6.80 (6.08–7.90)	<0.001
Blood glucose (mmol/L)	4.84 ± 0.27	6.89 ± 0.56	6.53 ± 0.43	0.017
HDL-C (mmol/L)	1.08 ± 0.07	1.11 ± 0.07	0.96 ± 0.05	0.179
LDL-C (mmol/L)	2.80 ± 0.18	2.92 ± 0.22	2.44 ± 0.17	0.160
Creatinine (umol/L)	76.53 (66.71–85.24)	57.30 (49.40–67.60)	185.55 (127.57–360.85)	<0.001
BUN (mmol/L)	5.54 (4.57–6.82)	5.90 (5.10–6.85)	11.90 (9.50–19.63)	<0.001
TC (mmol/L)	4.70 ± 0.16	4.79 ± 0.23	4.67 ± 0.23	0.935
TG (mmol/L)	1.40 ± 0.33	1.68 ± 0.19	2.04 ± 0.15	0.089
eGFR (CKD-EPIml·min-1·(1.73m2)-1)	101.00 (93.00–113.00)	104.00 (99.50–115.50)	35.50 (12.75–53.00)	<0.001
PCR	0.08 (0.06–0.11)	0.05 (0.04–0.09)	3.24 (0.71–5.82)	<0.001
ALP (U/L)	98.97 ± 6.39	67.44 ± 3.83	67.44 ± 7.77	0.001
Albumin (g/L)	42.77 (41.97–44.72)	38.80 (37.25–43.10)	33.55 (29.75–39.30)	<0.001
γ-GT(U/L)	22.82 (13.39–80.07)	20.00 (13.85–27.20)	21.35 (12.25–38.33)	0.878
Globulin(g/L)	27.23 ± 0.96	22.99 ± 0.73	26.43 ± 0.75	0.004
NLR	2.51 (1.83–3.09)	2.38 (1.97–3.04)	2.54 (2.09–3.37)	0.527
PLR	123.32 ± 8.49	122.30 ± 10.68	143.64 ± 8.80	0.184
LMR	3.62 (2.55–5.38)	5.32 (3.58–6.67)	3.05 (2.41–4.21)	0.001
ACR	2.19 (0.68–4.55)	2.96 (1.58–4.05)	1210.97 (158.64–3109.32)	<0.001
24-h urine protein(g/d)	–	–	2.78 (0.71–6.74)	–
MHR	0.50 ± 0.05	0.31 ± 0.05	0.60 ± 0.06	0.003

Measurement data were expressed as mean ± standard deviation (SD) or median and interquartile range according to the normal distribution statue, categorial data were expressed as numbers and percentages.

BMI body mass index, HbA1c glycated hemoglobin, HDL-C high density lipoprotein-cholesterol, LDL-C low density lipoprotein-cholesterol, BUN blood urea nitrogen, CKD-EPI The Chronic Kidney Disease Epidemiology Collaboration, TC total cholesterol, TG triglycerides, PCR Urine protein to creatinine ratio, ALP Alkaline phosphatase, γ-GT γ-Glutamyl transpeptidase, NLR Neutrophil to lymphocyte ratio, PLR Platelet to lymphocyte ratio, LMR Lymphocyte to monocyte ratio, ACR Albumin to creatinine ratio, MHR monocyte to HDL ratio.

**Table 2.** Expression levels of miRNA-615-3p and miRNA-3147 in urinary exosomes.

miRNA	*N*	T2DM	DKD	*P*-value
	(*n* = 20)	(*n* = 21)	(*n* = 42)	
miRNA-615-3p	0.16	0.62	1.34	<0.001
	(0.04–0.62)	(0.17–1.44)	(0.68–5.15)	
miRNA-3147	1.24	1.60	1.98	0.419
	(0.33–2.89)	(0.37–3.19)	(0.82–4.14)	

### Correlation of urinary exosomal miRNA-615-3p and miRNA-3147 with clinical indicators of DKD

**Figure 3.**
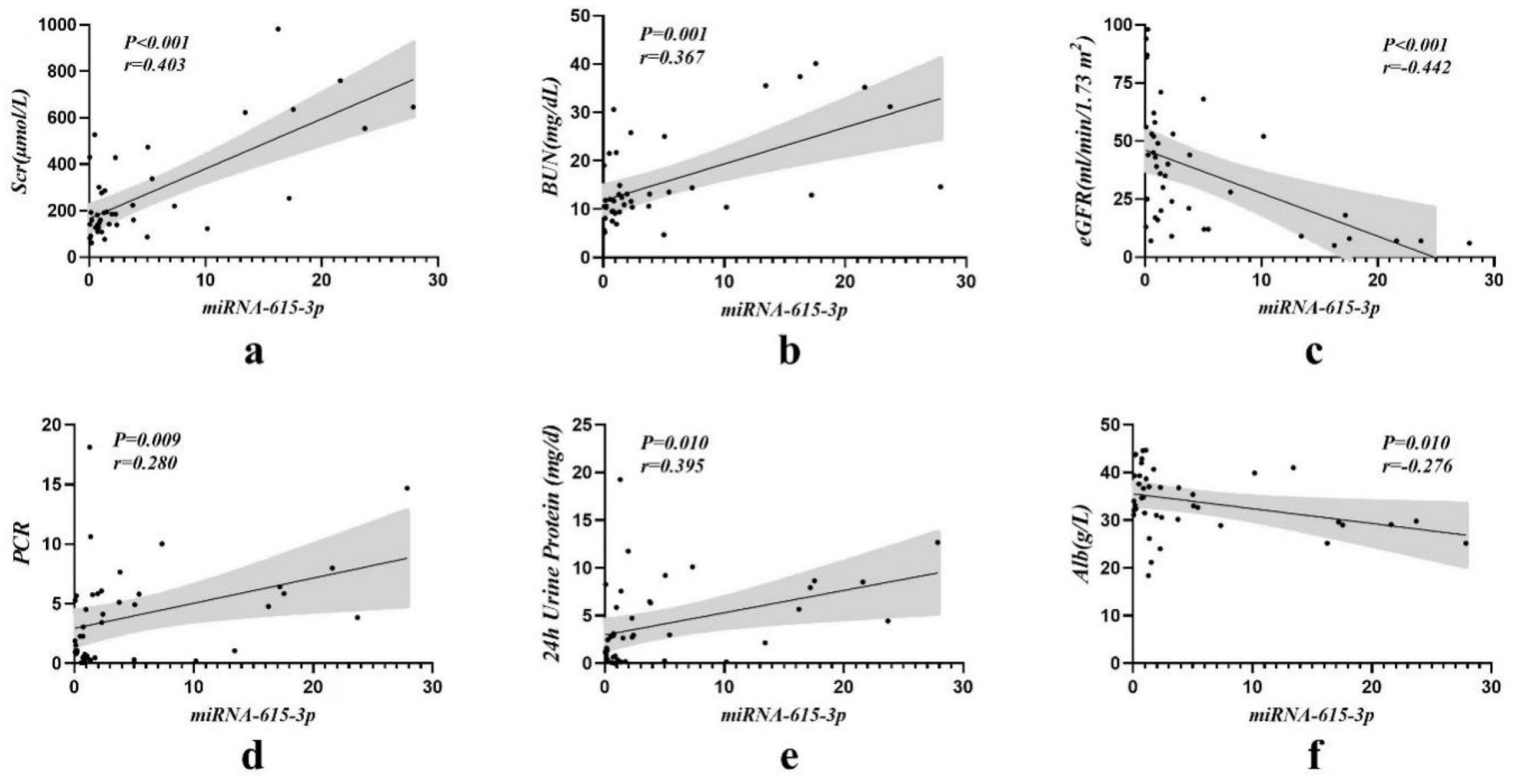
Correlation analysis of urinary exosomal miRNA-615-3p with clinical indicators in DKD group. (a) Scr. (b) BUN. (c) eGFR. (d) PCR. (e) 24 h urine protein. (f) Albumin.

We further investigated the correlation between the expression levels of the two miRNAs in urinary exosomes and clinical parameters (shown in Table 3 and Figure 3). The results showed that the expression level of urinary exosomal miRNA-615-3p was positively correlated with serum creatinine (Scr), urea, protein to creatinine ratio (PCR) and 24-h urinary protein, and negatively correlated with estimated glomerular filtration rate (eGFR) and albumin, all of which were statistically significant (*p* < 0.05). There was no significant correlation with other clinical indicators. However, urinary exosomal miRNA-3147 was only positively correlated with triglycerides (*p* = 0.029). In addition, although there were differences in age levels between the control and DKD groups, this parameter was not significantly associated with urinary exosomal miRNA-615-3p or miRNA-3147.

**Table 3.** Correlation analysis of urinary exosomal miRNA-615-3p and miRNA-3147 with clinical indicators in DKD group.

Clinical parameters	miRNA-615-3p		miRNA-3147	
	*r*-value	*P*-value	*r*-value	*P*-value
Age (years)	0.006	0.957	0.040	0.799
Gender (male, %)	0.141	0.274	0.250	0.111
BMI	0.015	0.888	−0.053	0.739
Mean arterial pressure (mmHg)	0.022	0.837	0.143	0.367
HbA1c (%)	−0.139	0.200	−0.093	0.558
Blood glucose (mmol/L)	0.067	0.530	−0.079	0.619
HDL-C (mmol/L)	0.107	0.319	−0.079	0.621
LDL-C (mmol/L)	0.005	0.965	−0.170	0.282
Creatinine (umol/L)	0.403*	<0.001*	0.018	0.910
BUN (mmol/L)	0.367*	0.001*	−0.015	0.925
TC (mmol/L)	0.037	0.729	−0.070	0.660
TG (mmol/L)	−0.024	0.820	0.338*	0.029*
eGFR (CKD-EPIml·min-1·(1.73m2)-1)	−0.442*	<0.001*	−0.086	0.588
PCR	0.280*	0.009*	−0.061	0.701
ALP (U/L)	−0.125	0.246	−0.001	0.994
Albumin (g/L)	−0.276*	0.010*	0.075	0.637
γ-GT(U/L)	−0.088	0.410	−0.068	0.670
Globulin(g/L)	−0.091	0.398	0.063	0.694
NLR	0.136	0.205	−0.019	0.904
PLR	0.168	0.116	0.022	0.891
LMR	−0.062	0.566	0.071	0.657
ACR	0.193	0.221	−0.127	0.422
24-h urine protein(g/d)	0.395*	0.010*	−0.059	0.713
MHR	−0.078	0.468	−0.064	0.688

We divided the DKD patients into 5 stages according to the level of eGFR: G1 + G2 stage (>60 mL·min-1·(1.73m^2^)^−1^), G3 stage (30–59 mL·min-1·(1.73m^2^)^−1^), G4 stage (15–29 mL·min-1·(1.73m^2^)^−1^) and G5 stage (<15 mL·min-1·(1.73m^2^)^−1^). The expression levels of urinary exosomal miRNA-615-3p relative to the stages of DKD were analyzed (shown in Table 4), and the expression level of urinary exosome-derived miRNA-615-3p gradually increased with the progression of DKD disease which was statistically significant between the DKD stages (*p* = 0.009).

**Table 4.** Urinary exosomal miRNA-615-3p in patients with different stages of DKD.

Clinical parameters	Number of subjects (%)	Expression of miRNA-615-3p	*P*-value
Gender			0.284
Male	28 (66.67%)	1.13 (0.60–4.75)	
Female	14 (33.33%)	2.00 (0.94–8.36)	
Stage (ml·min-1·(1.73m2)-1)			0.009
G1 + G2 (>60)	7 (16.67%)	0.22 (0.12–1.34)	
G3 (30–59)	16 (38.09%)	1.03 (0.72–1.90)	
G4 (15–29)	8 (19.05%)	1.82 (0.91–6.44)	
G5 (<15)	11 (26.19%)	13.42 (2.28–21.60)	

### Correlation analysis of urinary exosomal miRNA-615-3p and inflammation-related indicators

**Figure 4.**
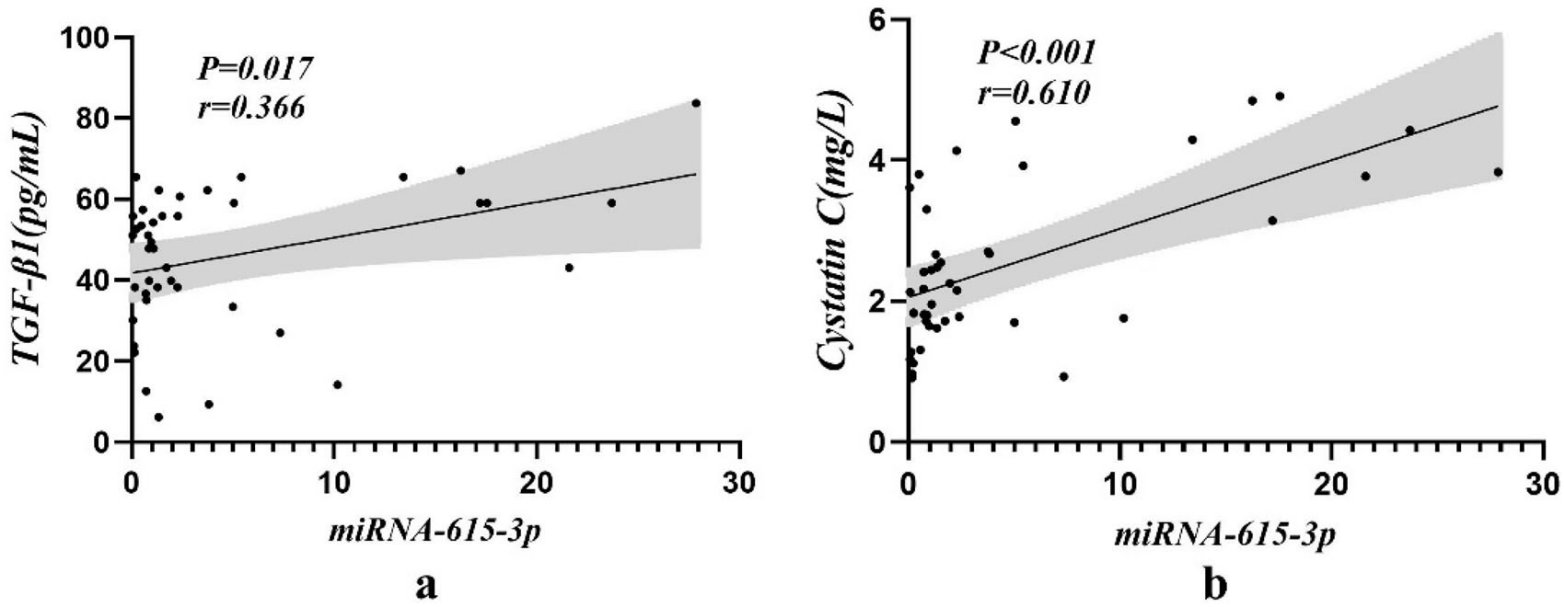
Correlation of urinary exosomal miRNA-615-3p with serum Cystatin C and TGF-β1 in DKD group. (a) Cystatin C-miRNA-615-3p. (b) TGF-β1- miRNA-615-3p.

The concentrations of plasma TGF-β1 (by ELISA) and serum Cystatin C (an inflammatory response molecule) were compared with the differentially expressed urinary exosomal miRNA-615-3p (shown in Table 5 and Figure 4). The results demonstrated that the expression levels of serum Cystatin C and urinary exosomal miRNA-615-3p were significantly positively correlated in the DKD patients (*p* < 0.001). Similarly, the expression levels of TGF-β1 and urinary exosomal miRNA-615-3p were significantly positively correlated in the DKD patients (*p* = 0.017).

**Table 5.** Correlation of serum Cystatin C and TGF-β1 expression levels with miRNA-615-3p in DKD patients.

Clinical parameters	Cystatin C		TGF-β1	
	*r*-value	*P*-value	*r*-value	*P*-value
miRNA-615-3p	0.610	<0.001	0.366	0.017

### Diagnostic value of urinary exosomal miRNA-615-3p and miRNA-3147 in DKD

**Figure 5.**
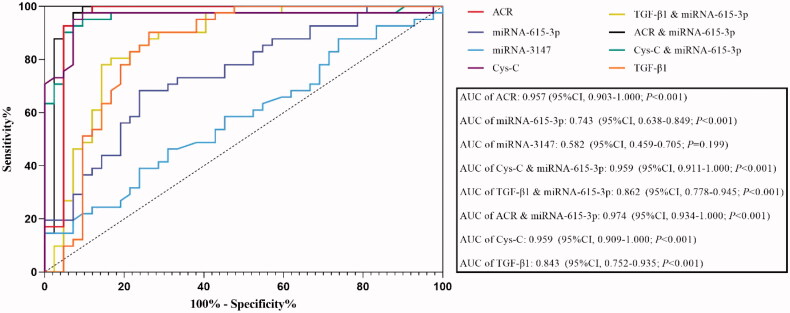
ROC curve analysis results of patients in the DKD group.

We used the ROC curve to analyze the diagnostic value of urinary exosomal miRNA-615-3p and miRNA-3147 individually in the diagnosis of DKD, and miRNA-615-3p combined with Cystatin C, TGF-β1 and ACR, respectively, in the diagnosis of DKD. The intent was to determine if the miRNAs individually or in combination with the other markers could improve the sensitivity and specificity of the DKD diagnosis. The ROC curve results revealed that urinary exosomal miRNA-615-3p exhibited an AUC of 0.743 (95%CI, 0.638–0.849, *p* < 0.001), whereas urinary exosomal miRNA-3147 exhibited an AUC of 0.582 (95%CI, 0.459–0.705, *p* = 0.199) (shown in Figure 5). The AUC values of albumin-to-creatinine ratio (ACR) in the DKD group were 0.957 (95%CI, 0.903–1.000; *p* < 0.001). The AUC for urinary exosomal miRNA-615-3p respectively combined with Cystatin C, TGF-β1 and ACR were 0.959 (95%CI, 0.911–1.000; *p* < 0.001), 0.862 (95%CI, 0.778–0.945; *p* < 0.001) and 0.974, (95% CI, 0.934–1.000; *p* < 0.001). However, the AUC values of Cystatin C and TGF-β1 in the DKD group were 0.959 (95%CI, 0.909–1.000; *p* < 0.001) and 0.843 (95%CI, 0.752–0.935; *p* < 0.001). Thus, urinary exosomal miRNA-615-3p combined with Cystatin C and ACR, respectively, had a higher diagnostic value for DKD than the traditional index ACR.

## Discussion

Exosomes are a special class of extracellular vesicles, a heterogeneous group of cell-derived membrane structures [23]. They originate from the endosomal system or the plasma membrane and can be released by budding from the plasma membrane of the donor cell [24]. They contain a number of molecules such as proteins, DNA, RNA, and lipids [25]. Exosomes can bind to recipient cells through plasma membrane fusion or endocytosis, and participate in intercellular communication [26]. These features make exosomes indispensable links in various pathophysiological mechanisms, enabling investigation of the pathogenesis of diseases to be studied at the cellular and molecular levels. Exosomes and the signal molecules they carry are, therefore, linked with the occurrence and development of corresponding diseases, such as tumors [27,28], idiopathic pulmonary fibrosis [29], atherosclerosis [30], immune disease [31–33] and certain metabolic diseases [34]. The development and progression of DKD are also closely related to exosomes. Under the stimulation of high glucose and oxidative stress, glomerular endothelial cells release TGF-β-rich exosomes, which interfere with the signaling pathway between endothelial cells and podocytes, thereby injuring podocytes [35]. Through glomerular-tubular signaling, the p53 pathway is induced in renal tubular cells, leading to renal tubular injury and promoting the generation of renal interstitial fibrosis [36]. Although the pathogenesis of DKD has not been fully elucidated, numerous reports have indicated that exosomes are involved in the pathophysiological onset of DKD, and are expected to serve as a novel biomarker for early diagnosis of DKD. Urine is produced by renal tubular reabsorption after passing through the glomerular filtration barrier, and exosomes secreted by glomerular and tubular cells are released into the urine. Therefore urinary exosomes are more likely to reflect kidney dysfunction than serum exosomes [37]. Therefore, urine is a convenient, high-quality source for biomarkers of kidney disease [38]. In the present study, urinary exosomes were selected as the basis for further experiments.

As a novel metastasis-related miRNA, miRNA-615-3p has been principally investigated relative to tumor diseases in recent years. High expression levels of miRNA-615-3p have been shown to directly affect several tumor target genes, such as breast cancer [39], small cell lung cancer [40]、prostate cancer [41], and function to regulate tumor cell growth. miRNA-615-3p plays a role in regulating the expression of insulin-like growth factor-2 (IGF-2) by binding to the 3′-UTR region of the gene. The occurrence and development of early DKD have been confirmed to involve IGFs [42,43]. In the pathogenesis of osteoarthritis, miRNA-615-3p was reported to participate in the inflammatory response related to osteoarthritis by increasing the expression of inflammatory cytokines, such as interleukin-1, interleukin-6 and interleukin-α [44]. It is well known that inflammatory response is also an important part of the pathogenesis of DKD. Therefore, combined with the results of this experiment, urinary exosomal miRNA-615-3p may be involved in the disease progression of DKD, but the specific mechanism will require further study. Nevertheless, many studies have indicated that cytokines and the molecular signaling pathways they participate in provide a central link in related inflammation and fibrosis [45].

As a traditional inflammatory indicator, Cystatin C not only reflects the degree of renal damage but is also one of the important indicators of the micro-inflammatory state in DKD patients [46]. Cystatin C is a low molecular weight protein (13KD) produced by all nucleated human cells and is primarily catabolized by proximal tubular cells [47]. As an endogenous inhibitor of cysteine protein, it is not affected by age, gender, muscle mass, or protein intake [48]. However, studies [46] have shown that serum Cystatin C is positively correlated with the micro-inflammatory state of DKD and related inflammatory factors. Cystatin C is not only correlated with eGFR and other indicators of renal function damage but also in evaluating the degree and progression of inflammatory response in the course of DKD. The present study provided evidence that the expression level of urinary exosomal miRNA-615-3p correlated with serum Cystatin C, so urinary exosomal miRNA-615-3p might be useful as a marker of the inflammatory response and the progression of kidney damage in DKD.

TGF-β1 is also involved in the process of renal tissue fibrosis [49]. TGF-β1, a member of the transforming growth factor β family, is a polypeptide with a molecular weight of 25KD, which can be secreted by a variety of immune cells [50]. It can induce the expression of chemokine in the kidney and participate in the occurrence and development of an inflammatory response [51], and regulate the synthesis of extracellular matrix by increasing the expression of TGF-1 mRNA and related proteins in the kidney of patients under the stimulation of hyperglycemia [52]. TGF-β1 is closely related to the inflammatory and fibrotic response of DKD, and previous studies [53] have shown that miRNA-615-3p can participate in breast cancer progression by increasing the downstream signal transduction of type I receptor TGF-β. By comparing the expression levels of urinary exosomal miRNA-615-3p and plasma TGF-β1, we found that with an increase of miRNA-615-3p, the expression level of plasma TGF-β1 also increased. Therefore, urinary exosomal miRNA-615-3p might act as an important regulator in the molecular mechanism of DKD inflammation and fibrosis.

Renal biopsy is the gold standard for the diagnosis of DKD. However, its invasiveness [54] precludes its use for either routine screening or follow-up of diabetic patients. It is often used in the differential diagnosis of DKD and other kidney diseases. In contrast, clinical criteria are usually used to diagnose DKD, one of which is ACR. However, it is easily affected by factors such as urine concentration, exercise, hypertension, and infection, which can result in a false negative or false positive diagnosis [55]. The present study analyzed the diagnostic value of urinary exosomal miRNA-615-3p combined with serum Cystatin C, TGF-β1 and ACR in DKD, and the results suggested that the diagnostic value of serum Cystatin C and ACR for DKD was higher than that of traditional index ACR. Moreover, urinary exosomal miRNA-615-3p, with its notable stability, when combined with ACR, had the highest sensitivity for establishing the clinical diagnosis of DKD.

## Conclusion

Urinary exosomal miRNA-615-3p may become a novel noninvasive biomarker for evaluating the progression of DKD, which would enable earlier intervention to improve its clinical management. It might also further contribute to our understanding of the pathogenesis of DKD.

## Data Availability

The datasets used and/or analyzed during the current study are available from the corresponding author on reasonable request.
